# Loss of Tolerance in Long-Term Allergen Immunotherapy: A Case of Late-Onset Systemic Anaphylaxis

**DOI:** 10.7759/cureus.93510

**Published:** 2025-09-29

**Authors:** Kousha Ehsani, Kaveh Mozafari, Mohammadreza Davoudpour

**Affiliations:** 1 Family Medicine, Surgery, University of Toronto, Toronto, CAN; 2 Surgery, West Suburban Medical Centre, Oak Park, USA; 3 Medicine, St. George's University School of Medicine, St. George, GRD; 4 Family Medicine, TruCare Medical Clinic and Pharmacy, Richmond Hill, CAN

**Keywords:** allergen-specific immunotherapy (ait), anaphylactic reaction, drug allergen interaction, fluoxetine, immune reprogramming, immune tolerance loss, late-onset systemic reaction, post-injection monitoring, selective serotonin reuptake inhibitor (ssri)

## Abstract

This case report documents a rare phenomenon of a serious systemic allergic reaction following nearly five years of ongoing allergen immunotherapy (AIT) in a 19-year-old male patient. The patient, stable on maintenance AIT for sensitization to dust mite, cat dander, and birch pollen, developed acute respiratory distress minutes after a routine injection. Emergency treatment with epinephrine, diphenhydramine, and salbutamol was successful. This case highlights several potential causes, including loss of immune tolerance, immune deviation due to infection, and possible immunomodulating properties of concurrent fluoxetine treatment. It is a reminder that patients on long-term AIT need to continue on follow-up and that drug-AIT interactions are an area for ongoing investigation.

## Introduction

Allergen immunotherapy (AIT) is a well-proven and effective long-term treatment for immunoglobulin E (IgE)-mediated allergic conditions such as allergic rhinitis, asthma, and venom hypersensitivity [1]. AIT involves the administration of progressively increasing quantities of the offending allergen to induce immunological tolerance and reduce symptom severity [2]. The therapeutic mechanisms are desensitization of effector cells, induction of allergen-specific regulatory T cells (Tregs), enhanced production of blocking IgG4 antibodies, and a shift from Th2- to Treg-dominant immune profiles [3].

Although systemic reactions to AIT during the early and intermediate phases - typically within 30 minutes of the injection - are well documented, delayed or late reactions are less well recognized, particularly in patients who have exhibited long-term tolerance without a previous history of adverse reaction [4]. The existing guidelines emphasize the merit of dose escalation recommendations and post-injection observation windows to reduce risk; however, existing research does not explain why a reaction might be seen many years after stable dosing [5].

This case report describes a highly unusual occurrence of a systemic allergic reaction in a patient after more than four years of continuous AIT, from September 2020 to May 2025. The purpose of this discussion is to explore the potential mechanisms of late-phase responses, including immune reprogramming, infection-related immune alterations, and the potential for drug interference. In this way, this report will contribute to better, safer clinical practice and offer hypotheses for future research.

## Case presentation

A 19-year-old Middle Eastern male patient, of Iranian descent, presented to the clinic for a scheduled allergen immunotherapy injection. His medical history was remarkable for pilonidal sinus surgery, a history of allergies to non-steroidal anti-inflammatory drugs (NSAIDs), and psychiatric consults with medications being prescribed. 

In August 2021, a psychiatric consult was requested due to anxiety and depression running in the patient’s maternal family. The result was a diagnosis of obsessive-compulsive disorder (OCD) for which the patient was prescribed fluoxetine 20 mg/day. The dosage of this drug was then increased in June 2024 to 40 mg/day, on which he remained, up to and after the severe anaphylactic reaction in May 2025.

The pilonidal sinus surgery with skin shift and advancement flap was done in 2023, after which he experienced minor infections at the surgical site. The disease was treated with Keflex and Flagyl, commonly known as cephalexin and metronidazole respectively, for seven days. 

Before the commencement of allergy injections in September 2020, a skin prick test was conducted by an allergist to determine the patient’s environmental allergies. This method is widely used in determining type I hypersensitivity reactions [6]. Using saline as the negative control and histamine as the positive control, this method enables the accurate interpretation of skin prick test results. The results showed mild sensitization (+1) for the following allergens: dust mite (D.F.), dust mite (D.P.), birch, ragweed, and mulberry, while moderate sensitization (+2) was observed for cat (see Table 1). Table 1 provides a comprehensive interpretation of the skin prick test, demonstrating that the patient was tested against a wide range of environmental allergens, thereby reducing the possibility that an untested allergen triggered the anaphylactic reaction.

**Table 1:** Skin Prick Test Results after Interpretation

Allergen	Result
Saline	-
Histamine	3 mm
Alternaria	-
Asp. F	-
Clad	-
Penicillium	-
Mold mix	-
Rabbit	-
Hamster	-
Guinea pig	-
Dust mite (D.F.)	+1
Dust mite (D.P.)	+1
Cockroach	-
Mouse	-
Cat	+2
Dog	-
Horse	-
Feathers	-
Cedar	-
Pine	-
Birch	+1
Ash	-
Elm	-
Grass	-
Rye grass	-
Oak	-
Ragweed	+1
Weed mix	-
Hickory	-
Mulberry	+1

Thus, the allergist began the allergy injections, which were a mix of both kinds of mites to which the patient had shown a reaction in the skin prick test, cat dander, and birch tree pollen. The initial serum was composed of 2.00 mL of standardized mixed mites, 1.19 mL of birch mix, and standardized cat hair. The concentrations of each component were 10,000 AU/mL, 1:20 W/V, and 10000 BAU/mL, respectively. The first injection was administered on September 29, 2020, with a dosage of 0.05 cc. In July 2022, a new allergy serum with increased dosages of allergens was developed as part of the AIT treatment. The allergist regularly monitored the dose, and in the medical notes, mentioned that possible allergic symptoms, such as minor discomfort and swelling, were present; however, no cases of anaphylaxis occurred.

In addition to his long-term fluoxetine therapy, the patient was otherwise healthy at the time of the event in May 2025, with no recent infections, missing AIT doses, or new drugs. Since 2021, fluoxetine has been used to treat his OCD. In June 2024, the dosage was raised to 40 mg per day. On this regimen, he reported consistent mental control and good adherence. In light of recent research indicating that selective serotonin reuptake inhibitors (SSRIs) have moderate immunomodulatory effects, which will be discussed later on in this paper, this concomitant treatment is significant when examining potential causes of his late-onset systemic reaction. 

After four and a half years of regular, weekly, and incident-free AIT, the patient received his next injection, the same dosage as the week before, in May 2025 and was instructed to remain in the waiting area for 15 minutes. After seven minutes, he reported difficulty breathing. His respiration rate increased dramatically, as observed by the physician, and his oxygen saturation dropped below 90% and eventually down to the low 80s. There was audible wheezing, and as a result, the physician administered 50 mg of intramuscular diphenhydramine (Benadryl), 200 µg of salbutamol, and 0.3 mg of epinephrine (EpiPen). The patient’s condition improved, but he had complaints of being jittery. Emergency medical services were called, and the patient was taken to the hospital. The hospital’s diagnosis was an anaphylactic reaction, and the patient was discharged.

As a result of this incident, the patient's AIT plan was altered, and the dose was significantly lowered. He is now being monitored very closely with increased caution when increasing the dosage of the injections.

## Discussion

The data on anaphylactic reactions resulting from AIT are limited. There was, however, a recent study using the data from the European Anaphylaxis Registry that assessed AIT-induced anaphylaxis. This study found that 173 of 15,748 instances of anaphylaxis in the registry resulted from AIT. This equates to 1.1%. Additionally, the study revealed an increase in respiratory symptoms among specific demographics, particularly children and adolescents. This study concluded that there ought to be age-specific monitoring and appropriate emergency treatments [7].

While all previous research indicates that anaphylaxis is possible after AIT, there is no explanation for why this particular reaction occurred after five years of regular treatment and dosage increases. Specifically, why did the reaction occur when the dosage had not even changed from the previous week? Several possible explanations for AIT-related responses have been proposed in the published literature. This article will discuss each one individually; however, it is worth noting that the most plausible explanations fall under the umbrella of a shift in the immune system.

A shift in the immune system indicates a loss of tolerance. For reference, tolerance induced by AIT relies on a delicate balance of Tregs, blocking antibodies, IgG4, and an immune shift away from Th2 responses. If this balance is disrupted, the patient may become re-sensitized and react again. This disruption can be due to waning IgG4 levels or a decrease in Treg function [8]. Several environmental factors can contribute to this disruption. The first is irregular dosing of AIT, including skipped doses, extended breaks, or improper restarting of the regimen. This irregular dosing can lead to a decline in Treg activity and a reduction in IgG4 levels, both of which can alter immune responsiveness and counteract the effects of AIT [9]. This explanation does not apply to this patient since there was no irregular dosing. 

The following possible explanation is known as natural immune aging, also referred to as immune modulation. It has been found that Treg functions can decline over time as individuals age. A proposition is that immune reprogramming (such as AIT) during adolescence or pregnancy can also shift the immune profiles and reawaken Th2 pathways. Their reawakening can increase the risk of systemic reactions and the loss of allergen tolerance [3]. Immune reprogramming during adolescence can potentially explain the patient’s response; however, this process requires more time than one week, and the sudden reaction with no previous instances makes this explanation incomplete and unsatisfactory [3].

Viral or bacterial infections have also been proposed as a potential reason for a sudden reaction to regular AIT dosages. A study published in 2011 suggested that infections can trigger inflammatory cytokines, such as IL-6, IL-1β, and TNF-α, that suppress Treg function or potentially re-activate Th2 cells. This will directly result in a disruption of the delicate balance needed for desensitization. It has been observed that respiratory viruses can trigger a flare-up of allergic diseases in previously controlled patients [10]. This is a plausible explanation, given the patient's history of viral and bacterial infections.

There is one other possible explanation: medication interference. When the medical history of the patient is reviewed, it can be seen that the patient had a history of taking antibiotics for a previous surgery and fluoxetine, which his psychiatrist prescribed. According to current studies and information available, there are no established causal relationships between antidepressants such as fluoxetine and AIT, but there are some possible connections that should be considered. Drugs like fluoxetine are SSRIs, and SSRIs have mild immunomodulatory effects. The effects may include the modulation of T cell function or a reduction in pro-inflammatory cytokines. It should be noted that the T cell function modulation is not specific to Tregs or Th2, which are key for AIT [11]. These associations are not conclusive and only point to areas of science that require more research.

The potential implications for practice that this case study has are the reinforced importance of observation after the injection of an AIT dose. Additionally, the case raises the question of the importance of the other medication the patient is taking and whether there is a correlation, given the lack of adequate research. Therefore, this case suggests that the medications the patient is taking should be considered during the AIT treatment program, and dosage escalations should also be closely monitored.

The purpose of this case report is to present the case in as much detail as possible, allowing future researchers to utilize it for the betterment of science.

## Conclusions

This case presents a rare phenomenon: the occurrence of a systemic allergic reaction years after stable AIT. Even though the patient's therapy history, including daily dosing and allergen profile, ruled out common culprits such as dose escalation or missed injections, several probable mechanisms presented themselves. These included immune reprogramming during adolescence, viral or bacterial infections that induce inflammatory responses, and the theoretical but conceivable immunomodulatory effects of SSRIs, such as fluoxetine. The case highlights the critical role of post-injection monitoring, even in chronic patients, and raises concerns about whether current and past medications should be more officially integrated into AIT protocols. As the field of allergy progresses, an individualized case report like this can aid in identifying trends, confirming treatment, and enabling more tailored treatment.
